# Supplementary material for: Utilization of technology to provide on-the-job trainings on Emergency Obstetric and Neonatal Care: Perspectives of nurses and midwives working in Rwanda’s remote health facilities
Source: PLoS One. 2024 Apr 26;19(4):e0291219. doi: 10.1371/journal.pone.0291219 (PMC11051650; doi:10.1371/journal.pone.0291219)
Supplement: S1 File — (DOCX) [file pone.0291219.s002.docx]

### Demographic characteristics of the respondents

The study involved 149 health care providers (HCP) participated in the study. Of them 64.4% were female and 33.6% were male. The highest proportion of the HCP who participated in the study were less than 30 years of age (25.5%). The highest proportion of the participants were nurses 69.8%, while the remaining 30.2% were midwife. Among the nurses, 20.2% of them had high school diploma, 71.1% had advanced diploma, while 8.7% had a bachelor’s degree. Among the midwife, 93.3% of them had advanced diploma and 6.7% had bachelor’s degree. The highest proportion of the HCP who participated in the study had been working in maternity services for more than 5 years (64.4%). The detailed results are shown in Table 1 below.

**Demographic characteristics**

| 1. Sex of the respondents [n (%)] | | |
| --- | --- | --- |
| Female | 99 (64.4%) | |
| Male | 50 (33.6%) | |
| 1. Age group [n (%)] | | |
| Less 30 years | 38 (25.5%) | |
| 30-34 years | 25 (16.8%) | |
| 34-39 years | 31 (20.8%) | |
| 40-44 years | 29 (19.5%) | |
| 45-49 years | 15 (10.1%) | |
| 50 + years | 11 (7.4%) | |
| 1. Professional qualification [n (%)] | | |
| Nurse | 104 (69.8%) | |
| Midwife | 45 (30.2%) | |
| 1. Educational level [n (%)] | | |
| Nurse (n=104) |  | |
| High school diploma (A2) | 21 (20.2%) | |
| Advanced diploma (A1) | 74 (71.1%) | |
| Bachelor’s degree (A0) | 9 (8.7%) | |
| Midwife (n=45) |  | |
| Advanced diploma (A1) | 42 (93.3%) | |
| Bachelor’s degree (A0) | 3 (6.7%) | |
| 1. Years of experience in maternity services [n (%)] | | |
| 1 year or less | 17 (11.4%) | |
| Between 1 and 3 years | 29 (19.5%) | |
| Between 3 and 5 years | 7 (4.7%) | |
| More than 5 years | 96 (64.4%) | |
| 1. Type pf Health facility visited | | |
| DH | | 4 (10%) |
| HC | | 35 (87.6%0 |
| HP | | 1 (2.4%) |
