# Supplementary material for: Utilization of technology to provide on-the-job trainings on Emergency Obstetric and Neonatal Care: Perspectives of nurses and midwives working in Rwanda’s remote health facilities
Source: PLoS One. 2024 Apr 26;19(4):e0291219. doi: 10.1371/journal.pone.0291219 (PMC11051650; doi:10.1371/journal.pone.0291219)
Supplement: S1 Appendix — (DOCX) [file pone.0291219.s003.docx]

## APPENDIX 3: Randomly Selected facilities

|  | **Province** | **District** | **Sub-District** | **Health facility** |
| --- | --- | --- | --- | --- |
| 1 | Kigali City | Gasabo District | Kibagabaga Sub District | Gatsata CS |
| 2 | South | Muhanga District | Kabgayi Sub District | Musange HP |
| 3 | South | Nyamagabe District | Kaduha Sub District | Buruhukiro CS |
| 4 | North | Gakenke District | Ruli Sub District | Gihinga HP |
| 5 | North | Burera District | Butaro Sub District | Rugengabali HP |
| 6 | West | Nyamasheke District | Kibogora Sub District | Ruheru (kanjongo Nyamasheke) CS |
| 7 | South | Muhanga District | Kabgayi Sub District | Shyogwe CS |
| 8 | West | Rusizi District | Gihundwe Sub District | NYAMUZI HP |
| 9 | West | Karongi District | Kibuye Sub District | Manji HP |
| 10 | North | Rulindo District | Kinihira Sub District | Mubuga (Rulindo) HP |
| 11 | North | Gakenke District | Ruli Sub District | Huro HP |
| 12 | Kigali City | Gasabo District | Kibagabaga Sub District | Kidashya HP |
| 13 | West | Nyamasheke District | Kibogora Sub District | JARAMA HP |
| 14 | West | Nyabihu District | Shyira Sub District | Birembo CS |
| 15 | West | Ngororero District | Kabaya Sub District | Hindiro CS |
| 16 | South | Gisagara District | Kibilizi Sub District | Mugombwa CS |
| 17 | North | Gakenke District | Ruli Sub District | Ruli DH |
| 18 | South | Gisagara District | Kibilizi Sub District | Kibilizi (gisagara) CS |
| 19 | West | Rubavu District | Gisenyi Sub District | Busasamana CS |
| 20 | North | Gakenke District | Nemba Sub District | Nemba DH |
| 21 | East | Nyagatare District | Nyagatare Sub District | Nyakigando (Nyagatare) CS |
| 22 | West | Rusizi District | Gihundwe Sub District | Gihundwe CS |
| 23 | East | Bugesera District | Nyamata Sub District | Ntarama CS |
| 24 | South | Kamonyi District | Remera Rukoma Sub District | Mugina HP |
| 25 | East | Gatsibo District | Kiziguro Sub District | Bugarura CS |
| 26 | East | Kirehe District | Kirehe Sub District | Mahama CS |
| 27 | East | Ngoma District | Kibungo Sub District | Karembo HP |
| 28 | North | Burera District | Butaro Sub District | Kamanyana HP |
| 29 | West | Nyabihu District | Shyira Sub District | Shyira CS |
| 30 | South | Ruhango District | Ruhango Sub District | Kigoma CS |
| 31 | East | Nyagatare District | Nyagatare Sub District | MBALE HP |
| 32 | East | Bugesera District | Nyamata Sub District | Ramiro HP |
| 33 | South | Gisagara District | Kibilizi Sub District | Kibilizi DH |
| 34 | South | Gisagara District | Gakoma Sub District | Gakoma DH |
| 35 | Kigali City | Kicukiro District | Masaka Sub District | Masaka HP |
| 36 | East | Nyagatare District | Nyagatare Sub District | Nkoma HP |
| 37 | West | Rutsiro District | Murunda Sub District | GITWA (MUSHUBATI) HP |
| 38 | Kigali City | Gasabo District | Kibagabaga Sub District | Kinyinya CS |
| 39 | West | Nyamasheke District | Bushenge Sub District | SAVE HP |
| 40 | South | Nyaruguru District | Munini Sub District | Rubona (Ngoma) HP |
