# Supplementary material for: Utilization of technology to provide on-the-job trainings on Emergency Obstetric and Neonatal Care: Perspectives of nurses and midwives working in Rwanda’s remote health facilities
Source: PLoS One. 2024 Apr 26;19(4):e0291219. doi: 10.1371/journal.pone.0291219 (PMC11051650; doi:10.1371/journal.pone.0291219)
Supplement: Attachment — Submitted filename: Response to reviewers.docx [file pone.0291219.s005.docx]

**Thierry Claudien Uhawenimana (MPHL, PhD)	29^th^ December 2023**

**University of Rwanda,**

**College of Medicine and Health Sciences**

**Po.Box:3286 Kigali Rwanda**

**Email:** [**tcuhawenimana@gmail.com**](mailto:tcuhawenimana@gmail.com)

**Phone: +250788586768**

**To: Editor**

**Plos One Journal**

**Re: Response to the editor’s and reviewers’ comments**

Dear Editor,

I am writing to express my gratitude on behalf of my co-authors for considering our manuscript and providing us with the opportunity to enhance its quality to meet the standards of your esteemed journal. We would like to extend our sincere appreciation to the editors and peer-reviewers for their valuable feedback. We have carefully considered their comments and incorporated the necessary revisions in the updated version of our manuscript, which is outlined in the table below.

Thank you once again for your support and consideration.

Sincerely,

Dr. Thierry Claudien Uhawenimana

| **Reviewers’ and editors’ comments** | **Response to the comments** |
| --- | --- |
| ***Academic editor*** | |
| Please ensure that your manuscript meets PLOS ONE's style requirements, including those for file naming. | Thank you for the observation. In the revised manuscript we formatted the paper according to the PLOS ONE style templates. |
| We suggest you thoroughly copyedit your manuscript for language usage, spelling, and grammar. | Thank you for this observation. In the revised version, we did our best to address some issues related to language usage, typos, and grammar. |
| Financial disclosure | We have indicated in the manuscript on the preliminary pages that the authors received no specific funding for this work. |
| We note that you have indicated that data from this study are available upon request. PLOS only allows data to be available upon request if there are legal or ethical restrictions on sharing data publicly. | We greatly appreciate you bringing up this matter. Sharing our data publicly does not violate any legal or ethical regulations. Specifically, the data we are referring to are the voice transcripts, which were submitted to UNFPA after undergoing thorough data analysis. If there is a requirement for the transcripts, we can request UNFPA to provide access to that data. |
| **Reviewer 1** | |
| Description of how objectives were achieved especially factors that may influence the application of this training approach in the Rwandan context is not clear. Mentioned that the study is a mixed method and elsewhere in the manuscript was mentioned as qualitative, need some clarification, otherwise the study sounds scientific with novelty of the subject. | Thank you for the observation. We carefully examined the methods section and rectified any typographical errors that could have led to the reviewer's confusion in categorizing our study as a mixed methods one. Furthermore, we have made necessary adjustments in the data analysis to accurately demonstrate the accomplishment of our study's objectives. For more information, please refer to the revised version of the manuscript, specifically pages 8-9. |
| **Reviewer 2** | |
| The introduction states: Furthermore, available research from Rwanda has focused on health facility managers’ perceptions on the use of e-learning in providing CPD for health providers’ capacity building purposes [20]. It would be helpful to revisit this in the discussion and understand how health facility managers' perspectives overlap or differ from that of nurses, midwives and EmONC mentors. | Thank you for this comment. In the discussion, we have discussed how nurses’ and midwives’ perspectives complemented those of the health facility managers in the study referred to in the introduction (see pages 16-17). |
| Consider providing demographic information about the participants, average years of experience with EmONC, level of education, previous experience with technological applications. This can help contextualize the findings. | Given that this study was conducted as part of the project 'USER RESEARCH FOR REMOTE CAPACITY BUILDING OF HEALTH CARE PROVIDERS ON EMONC IN RWANDA', it is important to note that we did not collect individual demographic information for participants in the qualitative phase. Nevertheless, we did provide demographic data for the participants in the quantitative study as a point of reference. |
| Please provide more detail on the coding process. How as the initial codebook developed? How many qualitative research experts coded each transcript? Did the qualitative research experts review and code together or independently? At final review, did the qualitative research experts discuss codes until achieving consensus? | Thank you for this important comment. We clarified the data analysis section to address some of the issues raised in this commentary. See page 9 for detail. |
| Theme 1: Access to EmONC CPD. This appears be less about access, with the predominant theme being perceptions on efficacy of in-person versus online training. Would suggest reframing the intro, moving the part about access to the moderators section (theme 3) and focusing on nurses’ perceptions of in-person versus online training. | Thank you for the observation. We considered the advice and had a theme titled ‘perceptions about in-person vs online training’ as theme 1 and the other content transferred to theme about the moderators. See pages 11, and 12-13. |
| “The common finding among all interviewees is that they appreciated the organizers of these CPD trainings, as they included the practical sessions as reported by one of the respondents.” Similarly, this may be less about appreciating the organizers and more about appreciation and need for practical, in-person trainings for specific topics.  “Participants expressed ambivalent views concerning the approach that can be used to on-job EmONC CPD courses.” With the majority preferring face-to-face trainings, it appears their views are similar and not ambivalent. | Thank you for this observation. We considered this commentary and addressed the slight confusion that occurred due to the choice of words we used. |
| Theme 2: Experience in using technology to access EmONC information. This theme is particularly brief. There may be value in revisiting the findings and assessing what can be added to this section. It may also be worth considering moving this to the moderators section, as it touches upon self-efficacy, digital literacy and health literacy. | We agree with the reviewer that the theme was brief and reflected the facilitators. So, we moved it to theme of the moderators. See page 14 in the revised manuscript. |
| Theme 3: Moderators of technology enhanced learning for EmONC CPD delivery. This section appears to be a mix of perceived benefits (access to video, audio, learning from work/no transport fees), needs (increase in the number of topics covered,) and moderators (staff motivation to enhance their knowledge and skills, accessibility: timing of live courses aligned with staff's free time/annual leave). Would suggest moving the benefits and needs to Theme 1: Perceptions of Online versus In-Person EmONC Trainings. Would suggest moving the challenges from Theme 1: insufficient skills in IT, lack of continued access to strong internet network to the moderators theme. | Thank you for the suggestion and we considered it and restructured the content of theme to keep it focused on the moderators. See pages 12-14 |
| financial benefits that may be associated with attending face to face trainings. Second, systemic factors: limited IT resources at the facilities and their readiness to adopt online learning approaches in building the capacity of their staff. It is unclear if these are findings of another study or this study. If this study, they appear to be new findings not previously stated. | Thank you for the observations. Those were findings from other studies and we added references to support those sentences. See page 17 |
| Given the number of focus groups and interviews conducted, it would be advisable to review the findings and offer a more robust analysis. While EmONC mentors where included, the majority of results focus on nurses and midwives. What were their views and how did they compare with nurses and midwives? Were there any difference in perceptions of staff from District Hospitals versus Health Centers and Health Posts? | Thank you for this comment. We did not carry out further analysis to compare the findings from focus group discussion and those from the key informant interviews because the purpose of our study was to garner nurses’ and midwives’ perspectives on the use of technology in providing EmONC CPDs in remote areas. Whilst it should be important to compare responses from both approaches to find out which one might be reliable and methodologically robust, we did not do that as our study was not a methodological efficacy one. Similarly, our study recruited participants who included nurses and midwives even the mentors were nurses and midwives who attended some trainings in EmONC. We therefore did not carry out a comparative analysis to compare the two groups because we had nurses, midwives, and mentors (who are nurses and midwives as well) in order to ensure the representativity of our sample not for the purposes of comparison. The same applies to district hospitals, health centers, and health posts. |
| **Reviewer 3** | |
| The title of the manuscript does not reflect the contents of the paper or rather the contents are not focused enough to be reflected in the title. Therefore either revise the title or (and that would be my suggestion) revise the manuscript to sharpen it to clearly address the key words from the title. | Thank you for the observation. We have revised the title to reflect the content of the paper. |
| Provide a clearer rationale for the research (rather than one which is alluded to indirectly) and Rwandan context (in as far as it related to the focus of the study presented in the manuscript). | We have revised the introduction to strengthen the rationale of our manuscript. See pages 4-5 |
| Provide a clear explanation for the e-learning platform being subject to the assessment in the research. There is a mention of the app being a new tool used for a short time under Limitations but really more information needs to be included in the earlier part of the manuscript. | Thank you for the observation. We have explained the e-learning tools that were subject for our manuscript. See page 5 |
| Provide more information on the study sites and respondents to allow the reader understand the methodology better. | Thank you for this observation. We have considered this comment and have provided information about the study sites and respondent. For details, we added a supplementary information to show where the study was conducted. |
| Assuming the data is reach in information, it may be useful to critically assess the themes to draw out a more nuanced and specific focus for the findings. Some of the aspects appear in multiple themes and overall the Findings section lacks a clear message - it 'jumps' from issues with IT skills and internet access, preference for face-to-face trainings, specific topics needed for training, then covers the strengths and positives of using e-learning, and other points all of which can build a more comprehensive picture if re-organised into a clearer narrative by the authors. | We have reorganized the results to remove a few confusions that affected their comprehension. |
| The discussion lacks inputs from a wider selection of other studies, which would be helpful for rooting the research conducted in a wider body of knowledge. | Thank you for this commentary. We have tried to add a few studies to expand the discussion of our findings. |
| Relating somewhat to the rationale, and the presentation of findings the Conclusions lack a more pragmatic focus, which would make the manuscript more useful for policy-makers and practitioners alike. Therefore it would be useful to sharpen the section to improve the reach the research may offer. | The conclusion was restructured to make it more relevant to the our study’s objectives and findings. |
| ‘Two focus group discussions with EmONC mentor, (…)’? -- should this say Two focus group discussions with EmONC mentors, (…)’? | Addressed. |
| ‘The major causes of these deaths are pregnant-related complications known as obstetric complications such severe bleeding and infections that occur after childbirth and abortion, (…)’ – should this say ‘The major causes of these deaths are pregnant-related complications known as obstetric complications such as severe bleeding and infections that occur after childbirth and abortion, (…)? | Addressed. |
| ‘One of the targets for the third sustainable development goals (SDGs) to reduce worldwide maternal mortality ratio (MMR) to (…)’ – should this say ‘One of the targets for the third sustainable development goals (SDGs) is to reduce worldwide maternal mortality ratio (MMR) to (…)’? | Addressed. |
| P4: ‘Technology enhanced learning has a number of benefits. First, it can mitigate some service delivery gaps that occur when nurses and midwives attend face to face trainings [13, 14]. Second, those who receive face to face trainings may not find it easy to transfer the skills they earn from such trainings [13, 14]. Third, some of the people who benefit from the face-to-face trainings may leave their places of work leaving personnel gaps [13, 14]. Fourth, technology enhanced learning are efficient because they can reach many people at a reasonable cost and at a regular time and meeting the pace and convenience of nurses and midwives [13, 14]. Lastly, need to shift from traditional way of learning new skills to the new way of acquiring skills using technological tools at nurses’ and midwives’ reach; promoting efficient and effective use of resources and also bolstering their capacity in digital health [15].': - Unclear: how does the observation that healthcare providers accessing face-to-face training can have problems with transferring skills relate to benefits of using technology enhances learning? Please elaborate. - How do points 1 and 3 differ? It seems in both the point is that due to in person attendance there are workforce gaps affecting service delivery. Please amend to make this clearer or if the two are in fact the same, please rewrite. - Also, these are limitations of in-person training, not benefits of technology-enhanced learning - please make the point more clearly. - The final sentence of the paragraph is not grammatically correct and therefore not very clear - please revise. | Thank you for pointing out this issue. In the current version, this content was removed. |
| P4: ‘In particular, e-learning and remote training is cost-effective compared to traditional learning methods and can provide space for easy scale-up and improvement of content availability [16].’: Is this not a benefit of e-learning technologies discussed in the above paragraph? Please consider including in the list above.  P4/5: ‘Moreover, e-learning has proven its relevance in teaching and learning process amidst COVID-19 pandemic, including during challenging times of movement restrictions and lockdowns[17].’ – as above. | Thank you for this comment. In the new version, we decided to remove this content to minimize overlaps. |
| P5: ‘Despite the progress made, less is known about how remote nurses and midwives are facilitated to attend CPDs. It is in this regard that the current study sought to test the feasibility of providing CPDs to nurses and midwives in hard-to-reach health facilities in Rwanda using user-friendly phone-based technology.’ And onwards: The Rwandan context requires some revising - while relevant points building the framework for where this study fits are provided, the specifics of the Rwandan MNH system and HCP training within that require more information to help the reader (who we should assume will have limited knowledge of it) understand the topic. | Thank you for this commentary. In the current version, we tackled the maternal and neonatal system for Rwanda. See page 5 |
| ‘Therefore, this study sought to understand from the nurses’ and midwives’ perspectives how technology-enhanced learning can be used to provide learner-centred CPD trainings for nurses and midwives about EmONC and factors that may influence the application of this training approach from the Rwandan context based on the newly developed EmONC smartphone application prototype.’: Overall the background assumes prior knowledge of the Rwandan context and 'jumps' between subtopics relevant as context to justify the need for the study. While potentially its benefits are understandable this section should be revised to build a clear case for the research while allowing the reader to learn more about the specific aspects of the healthcare system in Rwanda which make it interesting and relevant to focus on. | Thank you for this comment. We have addressed this comment in page 5-6 in the new version. |
| P6: Methodology: - Dates when the study was conducted are missing - please include. - This training programme / e-learning platform needs an introduction in the background section. | Dates when the study was conducted were added. Information about the technology platform discussed in the study has been introduced on page 5 of the revised manuscript. |
| ‘Data was collected from nurses and midwives from forty (40) public health facilities nationwide.’: How were these selected? How representative is the sample (if at all)? Out of how many overall? What type of facilities were included: lower/higher level, urban/rural, public/private, geographical spread, etc. Were the selected facilities part of a particular programme? Please include this information. This is somewhat addressed under Sample size and sampling strategy below, but it may be useful to reorganise the information to make it clearer. | Thank you for the observation. We have clarified our sample size determination and the sampling process. See page 7 for detail. |
| In total, 54 individuals were included in this study.’: What was the response rate? Were the same respondents in the FGDs and KIIs or was any individual only asked to participate in group discussions or interviews? | Thank you for the comment. Since our study was qualitative, we did not calculate the response rate. |
| Data collection process: What was the difference between the FGDs and KIIs data collection tools? Did they cover the same issues? | Thank you for the comment. The tool used moderating FGDs was also the one which was used to moderate key informant interviews. They covered same questions. |
| ‘We ensured that HCPs) felt (…)’ – please delete the superfluous bracket sign. | Thank you. The superfluous bracket sign was removed. |
| A thematic analysis was conducted and all five steps of qualitative-data analysis (reading, interpreting, coding, reducing, and displaying) were performed to ensure consistency within the data.’ - This sounds like steps from 'framework analysis' (Richie and Spencer) - is that the case? | Thank you. We indicated that we applied framework analysis by Richie and Spencer. |
| P10 and P13: ‘ptototype’ – please correct to ‘prototype’. | Addressed. |
| P10: Findings: In general, please revise the findings to make the arguments you are trying to convey clear - these also need to be substantiated by the data by including a quote or providing a feel for how common (or uncommon) a particular point was, and/or whether it was voiced unanimously or a particular group of respondents found it more relevant than others. | Thank you for the observation. We reorganized the findings to make arguments clear. See results section. |
| P10: Theme 1: Access to EmONC CPDs: There are two points re e-learning courses but they are not presented as such: 1. Online courses are more challenging to access due to required IT skills and poor Internet infrastructure; 2. Points awarded for e-learning being insufficient in comparison to the effort required for completing the training. | Addressed. |
| P10, P12 and elsewhere: please standardise the spelling of ‘face to face’ as either ‘face to face’ or ‘face-to-face’ as both forms are used in the manuscript. | Addressed. |
| P 10: HMS and HBB:– Please spell out the abbreviations and check for any other abbreviations which may not be explained. | Addressed. |
| P11: ‘on-job EmONC’ should read ‘on-the-job EmONC’. | Addressed. |
| P11: ‘The respondents mentioned that despite the usefulness of online CPD trainings particularly during emergencies such as Covid-19 pandemic, in-person trainings were perceived effective in equipping nurses and midwives with knowledge and translational practical skills in EmONC as illustrated in this quote.’: In more general terms the role online trainings in a CPD programme and how they could be improved to provide a more useful resource for healthcare providers is also covered in Theme 3 suggestion there is a link between sub-sections that may be better explored in the manuscript. | Thank you for the commentary. We have tried to explore that in the discussion. |
| P11: ‘However, some nurses and midwives; particularly (…)’ – should this read ‘However, some nurses and midwives, particularly (…)’? | Addressed |
| P12: Theme 3: Moderators of technology enhanced learning for EmONC CPD delivery: The point on IT skills was raised previously - could these be linked or combined? | Thank you for your observation. This observation corroborates reviewer’s suggestion, and we combined the two. |
| P13: ‘(…) it is difficult for us". One mentor mentioned.’ and ‘This is saddening to lose a mother or a baby." One midwife mentioned during the KIIs.’ - Standardise quotes: the follow up on one mentor mentioned coming after the quote does not follow the style used elsewhere. | Addressed. |
| P14: ‘During the discussions, nurses and midwives proposed to also avail an offline option of this prototype in order to mitigate the connectivity issue that may affect its functionality. Another barrier participants reported was financial constraint to access internet bundles and smartphones for some nurses and midwives, which can reduce their interest in using the application to learn.’: Internet access (as well as IT skills) have been raised earlier. In general, while the 'themes' cover specific topics, there is some overlap - whether that is a matter for reorganising information under each theme or providing clearer links this needs to be addressed to better guide the reader. | Thank you for this observation. We looked into the findings and reorganized them to reduce any redundancies. |
| P15: ‘Respondent said: (…)’ – should this read ‘One respondent said: (…)’? Please correct formatting to non-cursive for not-quote. | Addressed. |
| P15: Discussion: The discussion feels somewhat lacking in evidence from a wider body of literature. Please consider reading more broadly about online learning programmes, platforms and tools which may provide a helpful caparison for your research findings. | We considered this feedback and tried to add some evidence to support the findings. |
| P15: ‘Particularly, our study found that there is a need to provide refresher trainings about the management of pre-eclampsia.’: In fairness the focus on this particular topic does not come as strongly from the findings; eclampsia is mentioned alongside other topics flagged for future training ideas. If this was a particular critical point, please stress that in the findings. | Thank you for this observation. Given the context of Rwanda, though participants need trainings on other topics, but being trained on eclampsia and pre-eclampsia would contribute to maternal deaths that result from them (seeing that it is one of the leading causes of maternal morbidities and mortality in Rwanda). |
| P16: ‘Nurses and midwives are willing to undertaken (…)’ – should this read ‘Nurses and midwives are willing to undertake (…)’? | Addressed. |
| P16: ‘Nurses and midwives are willing to undertaken EmONC CPDs delivered through blended approach using both online approach and hands-on-skills physical trainings.’: This is a critical statement and I wonder whether this can be used better for fine-tuning the Findings section. The use of online training intuitively seems to be in-line with advances in technology and costs of in-person training but there are skills which cannot be learned by a phone app. Therefore it would be useful to understand from your study how the HCPs felt about e-learning in general, in terms of the specific app that they were using at present and how they would wish to see the use of e-learning going forward. What did they see as advantages, disadvantages and what the suggestions for making the platform better (more useful) were. This would provided a stronger analytical angle for policy and practice to draw out more specifically what works, what needs to improve and what the next steps ought to be. | Thank you for these insights. We though that if we use the statement ‘nurses and midwives are willing to undertake EmONC delivered through blended approach and hands-on-skills physical trainings’ would lead to overlaps particularly that the points the reviewer was making are covered in the findings. We acknowledge that in our study only focused about the findings that were about the perceptions of the newly developed and piloted technology tools. However, we do have findings about nurses’ and midwives’ perspectives about the advantages and the disadvantages of use of e-learning generally which we thought could not fit in the current manuscript. |
| P17: ‘Could they have got enough time to test and use the newly developed application and accessing the content of the application, the study could have garnered more insights on the feasibility of the developed application.’: This is a major point and really ought to have been raised as part of the introduction to the app to clarify exactly what was being used as a reference for the e-learning study! | The newly developed technological tools have been piloted in order to find out if there were any improvements needed. Since they were tested once during the covid-19 period, further te |
| P17: ‘Lastly, although the current study sought to explore how online and computer-based CPD delivery can be promoted to provide CPDs to nurses and midwives, it cannot be ignored that nurses and midwives need hand on skills practice to provide EmONC to the mothers and newborns hence a blended approach would always add value as highlighted by some respondents in this study.’: I am not sure this is a study limitation. Instead I think the context for the research needs to be set out more clearly to a priory specify that the nature of the practice of maternity staff requires practical skills which require in-person learning and practice and inline learning cannot be seen as a substitute for that. Instead the role of online training is to support, complement and enhance the learning process and as part of the study your findings have flagged what aspects make it a useful platform and where the shortcomings are. This could then be used by policy-makers to consider how to address the need for ongoing training (CPD) for maternity staff based on your work to incorporate what the evidence suggests should be continued, what should be developed further and what needs further thinking. This will make your research more useful for planning and informing policy and practice. | Thank you for this comment. We shifted that content into the conclusion. |
| P18 onwards: References: Spelling of WHO to be standardised in the Bibliography. | Addressed. |
