# Supplementary material for: Utilization of technology to provide on-the-job trainings on Emergency Obstetric and Neonatal Care: Perspectives of nurses and midwives working in Rwanda’s remote health facilities
Source: PLoS One. 2024 Apr 26;19(4):e0291219. doi: 10.1371/journal.pone.0291219 (PMC11051650; doi:10.1371/journal.pone.0291219)
Supplement: Attachment — Submitted filename: Rebutal letter to editors-Thierry et al.2024.docx [file pone.0291219.s006.docx]

**Point-by-point response to reviewers’ comments**

| **Comments** | **How the comments have been addressed** |
| --- | --- |
| **Academic Editor** | |
| Please review your reference list to ensure that it is complete and correct. If you have cited papers that have been retracted, please include the rationale for doing so in the manuscript text or remove these references and replace them with relevant current references. Any changes to the reference list should be mentioned in the rebuttal letter that accompanies your revised manuscript. If you need to cite a retracted article, indicate the article’s retracted status in the References list and also include a citation and full reference for the retraction notice. | *Thank you for your observation. Reference No6 (World Health Organization) was replaced with the most recent reference.* |
| ***Reviewer 2*** | |
| Write out full form of CPD at first use (abstract background) | *Addressed.* |
| Theme 1: Access to EmONC CPD. This appears be less about access, with the predominant theme being perceptions on efficacy of in-person versus online training. Access to CPD credits and internet is mentioned in the introduction, but not supported by quotes. If there are quotes supporting challenges in access, create a separate theme or consider adding to theme 3: Moderators of technology enhanced learning. | *Thank you for this observation. The comment was taken into consideration. See page 14-15* |
| Theme 3: The middle section (participants expressed that the number of topics they covered regarding EmONC was not sufficient) does not appear to be a moderator and is more related to acceptability (theme 4). | *Thank you for your observation. The comment was taken into consideration. See page 14-15* |
| ***Reviewer 3*** | |
| - As previously raised, on-job should be replaced by on-the-job | *Addressed throughout the paper.* |
| - As previously raised, please decide on the writing of ‘face to face’ and keep the consistency as either face-to-face or face to face | *Thank you for advising us to ensure consistency. We have decided to use face-to-face. See changes throughout the manuscript.* |
| Quotations need to be standardised – this has already been done but in one instance the quote does not follow the format (quote ‘Personally, as a midwife, (…)’), but also the way the quotes are presented could benefit from using a common style for indicating who the respondent was – normally after the quote you may see details such as profession, perhaps another identifier such as location (though ensuring this does not compromise the anonymity so perhaps a wider geographical region) and source of information (FGD, IDI) – with the relevant information in brackets immediately after the quote, instead of using text to describe the person as is currently applied. The quote would then read for example “xxxxxx” (Nurse, Y District, FGD) | *We have taken this comment into consideration and we made changes accordingly throughout the results section where there are quotes.* |
| - ‘Whatever complications that affect an expectant women experiences also causes serious effects to the baby’ – this reads somewhat clumsy – please revise | *We have rephrased the sentence to ensure clarity. See page 4* |
| - ‘This was done in order to find out if the newly design remote training tool (…)’ – should read ‘This was done in order to find out if the newly designed remote training tool (…)’ | *Thank you. The edit has been taken into consideration.* |
| ‘Qualitative Research Experts spelt also as Qualitative Research experts – please standardise and consider using lower case lettering for the term; | *The spelling issues have been addressed. See page 8* |
| Use of numbering (1 and 2) or letters (i and ii) to be applied for listing of themes – at present the first one is listed under ‘1’ and the second under ‘ii’. Also, the introduction to themes lists two but in the findings there are three themes, which do not match the ones provided under ‘Data analysis’ - please revise to make sure the text is correct | *The issue has been addressed. See page 9 for detail.* |
| As previously raised, flagging of pre-eclampsia under discussion does not match the findings; the explanation provided in the rebuttal letter needs to make its was into the manuscript to provide an explanation for why this particular aspect of emergency obstetric care is highlighted | *Thank you for your comment. We have discussed the findings in the context of other training needs participants expressed instead of focusing on pre-eclampsia only. See details on page 16* |
| There is no need to spell out EmONC after its first appearance in the text and use of the abbreviation alone is sufficient | *Addressed throughout the manuscript.* |
| - ‘Supplementary information 2’: The information in the supplementary file may need revision to ensure the files are clearly labelled, but also in the current document whatever is included under header ‘1.1.1’ and ‘Table 1’ needs relabelling as these numbers do not seem to match the document. Additionally, while providing data on the general population of the respondents is useful given the lack of similar data on the sample in the research study presented in the manuscript, the information needs to be suitably adapted to the manuscript; instead it reads as a copy from a general report which makes it challenging to follow as a reader – please revise to make it clearer. | *Regarding the issue raised in supplementary information 2, the authors have acknowledged in the manuscript the limitation of not including the sample characteristics for the qualitative component. However, sample characteristics of the broader project that contributed to the data provided in this manuscript have been presented.* |
