# Supplementary material for: Utilization of technology to provide on-the-job trainings on Emergency Obstetric and Neonatal Care: Perspectives of nurses and midwives working in Rwanda’s remote health facilities
Source: PLoS One. 2024 Apr 26;19(4):e0291219. doi: 10.1371/journal.pone.0291219 (PMC11051650; doi:10.1371/journal.pone.0291219)
Supplement: Attachment — Submitted filename: Nurses and Midwives Reviewer comments PLOSE ONE.docx [file pone.0291219.s004.docx]

**Nurses and Midwives' Perspectives on Technology-Enhanced Learning and Continuous Professional Development on Emergency Obstetric and Neonatal Care in Rwanda**

**Abstract section**

The abstract is well summarized

**Introduction section**

1. The introduction has good content however it is very long. Try to shorten it describing the issue globally, Africa, SSA, East Africa, locally, what was done so far and your main objective of your study
2. There some long hanging statements with no reference

**Methodology Section**

1. Study area: you better describe further your study setting( geographical location for each of your study setting, services provided in that setting in relation to your study and staff where applicable( Eg: providing deliveries and newborn care depending on level of facility you used)
2. Study design: -Indicate also your study duration

-Avoid reference in your study design section please

- Be concise and short. Avoid (Delete) your study objective in this	section

3. Study population: in your inclusion criteria, remove that statement from “regardless”. This is because you said you excluded nurses and midwives with less than 6 months of experience which is contradicting.
4. The sample size was relatively small to have some conclusions

You need to clarify more on how you determined your sample size. Tell us more on your saturation concept in relation to your study

5. Data collection procedure: who collected your data (be specific). Did train your research assistants
6. Data analysis: can you describe how you achieved your first and 2^nd^ objective? How did you determine factors that may influence the application of this training approach in the Rwandan context?
7. Results: how did you analyze factors influencing application of this training approach
8. There some few long hanging statements( without references)
9. Conclusion: is too long. it should be concise straight to point based on your results

No need of mentioning some of your results in this section please. Remove those results related statements.
